# Beta-Blockers After PCI for Stable Coronary Artery Disease and Preserved Left Ventricular Ejection Fraction

**DOI:** 10.1016/j.jacadv.2024.101566

**Published:** 2025-01-17

**Authors:** Safi U. Khan, Usman Ali Akbar, Muhammad Shahzeb Khan, Kershaw V. Patel, Amna Nadeem, Samarth Thakkar, Hassaan B. Arshad, Salim S. Virani, Khurram Nasir, Sachin S. Goel, Alpesh R. Shah, William Zoghbi, Neal S. Kleiman

**Affiliations:** a Department of Cardiology, Houston Methodist DeBakey Heart & Vascular Center, Houston, Texas, USA; b West Virginia University- Camden Clark Medical Center, Parkersburg, West Virginia, USA; c Division of Cardiology, Duke University School of Medicine, Durham, North Carolina, USA; d Department of Medicine, Punjab Medical College, Faisalabad, Pakistan; e Department of Medicine, The Aga Khan University, Karachi, Pakistan; f Baylor College of Medicine, Texas Heart Institute, Houston, Texas, USA

**Keywords:** beta-blocker, percutaneous coronary intervention, stable ischemic heart disease, target-trial emulation

## Abstract

**Background:**

Limited data exist on the long-term impact of beta-blocker therapy after percutaneous coronary intervention (PCI) in patients with stable coronary artery disease (CAD) and preserved left ventricular ejection fraction (LVEF).

**Objectives:**

The aim of the study was to evaluate the effects of early beta-blocker initiation vs no initiation following PCI in patients with stable CAD and preserved LVEF.

**Methods:**

This retrospective cohort study employed target trial emulation and incident user design, utilizing the TriNetx database (2009-2024). Early beta-blocker initiation (within days 1 and 7) was compared with no initiation using 1:1 greedy propensity score matching. The outcomes included all-cause mortality, hospitalization for myocardial infarction, heart failure, atrial fibrillation/flutter, stroke, and safety endpoints. Hospitalization for bone fracture and acute appendicitis served as falsification endpoints. In the intention-to-treat analysis, outcomes were analyzed over 5 years using Cox-proportional hazards.

**Results:**

Out of 11,681 matched patients per group, beta-blocker therapy was associated with increased all-cause mortality (HR: 1.11 [95% CI: 1.09-1.18]). No significant differences were found in hospitalization for myocardial infarction (HR: 1.03 [95% CI: 0.97-1.09]), stroke (HR: 0.98 [95% CI: 0.91-1.05]), heart failure (HR: 0.99 [95% CI: 0.95-1.03]), and atrial fibrillation/flutter (HR: 0.97 [95% CI: 0.93-1.01]). Hospitalization for hypotension was higher with beta-blockers (HR: 1.10 [95% CI: 1.06-1.14]). Hospitalization for bone fracture (HR: 1.02 [95% CI: 0.85-1.22]) and acute appendicitis (HR: 1.17 [95% CI: 0.95-1.45]) showed no significant associations. Several sensitivity analyses showed consistent results.

**Conclusions:**

Early beta-blocker initiation after PCI for stable CAD with preserved LVEF was associated with higher mortality, with no impact on cardiovascular events.

Beta-adrenergic receptor blockers have been a cornerstone in managing coronary artery disease (CAD) since early randomized controlled trials demonstrated their survival benefits after acute myocardial infarction (MI).^1^ Current American Heart Association/American College of Cardiology^2^ guidelines recommend beta-blocker use in patients with MI, those with left ventricular dysfunction (left ventricular ejection fraction [LVEF] of ≤50%) or systolic heart failure (HF) to mitigate the risk of cardiovascular events.^2^

Given the limited data, the current professional guidelines do not encourage the use of beta-blocker therapy after revascularization for stable CAD patients who do not have left ventricular dysfunction.^2^^,^^3^ However, a recent study revealed a reduction in primary composite endpoint (all-cause death and hospitalization for HF or MI; HR: 0.92 [95% CI: 0.86-0.98]), primarily driven by hospitalization for MI (0.87 [95% CI: 0.77-0.99]). Subgroup analysis for the primary endpoint showed no significant interaction based on percutaneous coronary intervention (PCI) within 90 days postindex (*P* for interaction = 0.49) and LVEF (*P* for interaction = 0.21).^4^

Prior observational studies were affected by several potential biases, such as confounding by indication^5^, where the clinical indication for the treatment may be inherently linked to the outcome; survivor treatment selection bias,^6^ which occurs when patients who live longer have more opportunities to receive treatment, leading to a fundamental difference between them and those who die earlier may remain untreated by default, immortal time bias;^7^ and prevalent user bias,^8^ significantly distorting the potential treatment effects of the therapy. Furthermore, skepticism exists in the scientific community regarding the likelihood of conducting a randomized trial to assess beta-blockade in stable CAD after PCI, considering the vast scale required for such a trial and the generic status of beta-blockers, which may diminish financial incentives for funding. In this context, we conducted a target trial emulation study to assess whether a strategy of early initiation of oral beta-blockade in patients with stable CAD and preserved LVEF after PCI influences all-cause mortality and cardiovascular outcomes and to evaluate the safety profile of this therapeutic strategy within this cohort.

## Methods

This study was exempt from institutional board review approval due to the use of deidentified publicly available data. This study follows the Reporting of studies Conducted using Observational Routinely-collected health data (RECORD) guidelines.^9^

### Study source

This study was conducted using the U.S. Collaborative Network of TriNetX database, a global federated health research network^10^ that facilitates access to electronic health records (EHRs), including demographic and socioeconomic status, diagnoses, and procedures (recorded using International Classification of Diseases-10th revision [ICD-10]) and Current Procedure Terminology codes), medications, laboratory values, and genomic data from more than 70 million patients across more than 60 health care organizations (HCOs) in the United States across 50 states, covering diverse geographic regions (22% Northeast, 16% Midwest, 39% South, 13% West, 10% unspecified). The TriNetX platform ensures that all displayed data or patient-level data provided in datasets are deidentified according to the standards outlined in Section §164.514(a) of The Health Insurance Portability and Accountability Act Privacy Rule. TriNetX aggregates and anonymizes electronic health record data from a network of HCOs, predominantly comprising large academic medical institutions with extensive inpatient and outpatient services spread across the United States. Self-reported sex (men, women) and race/ethnicity information within the TriNetX platform is derived from the electronic health record systems of the contributing HCOs; race is standardized into predetermined categories: Race (Asian, American Indian/Alaskan Native, Black/African American, Native Hawaiian/Other Pacific Islander, White, Unknown) and Ethnicity (Hispanic or Latino, not Hispanic or Latino, Unknown Ethnicity).

TriNetX conducts thorough data preprocessing to reduce missing values and standardizes the data into a uniform clinical model to ensure consistent query results across different data sources. All variables are structured as binary, categorical (converted to multiple binary columns), or continuous. Age data are always available. In cases where sex data is missing, it is labeled as “Unknown Sex.” Absences in race/ethnicity data are categorized as “Unknown Race” or “Unknown Ethnicity.” For other data types, such as medical conditions, procedures, lab tests, and social determinants of health, the data are either available or not; we excluded cases with missing values.

### Study design

This retrospective cohort study employed an incident user design^11^—a method that closely emulates randomized controlled trial—to examine the effects of the strategy of early initiation of beta-blocker therapy in patients undergoing PCI for stable CAD and those with preserved LVEF. This study approach includes only new treatment users to avoid biases related to previous treatment exposure and ensure a clearer temporal relationship between treatment initiation and outcomes. This design mitigates common biases such as confounding by indication,^5^ survivor treatment selection bias,^6^ immortal time bias,^7^ and prevalent user bias,^8^ by precisely defining the start of follow-up (time zero) when eligibility criteria are fulfilled and a treatment strategy is delineated for reasonable estimation of causal effects.

### Patients, treatment, and procedures

All analyses were performed between June 7 and July 14, 2024. Eligible patients were identified from a database spanning January 2009 to June 2024.^12^ The inclusion criteria focused on adult men and women (≥18 years) who underwent PCI for stable CAD with preserved LVEF (≥50%) (see Supplemental Table 1 for ICD codes). We excluded individuals who presented with acute coronary syndrome, who underwent PCI or coronary artery bypass graft (CABG) within the last 6 months, and who had any history of beta-blocker therapy use for any indication before the baseline assessment.

We defined stable CAD among patients who did not have acute coronary syndrome or received a revascularization procedure 6 months (washout period) prior to the study.

We constructed 2 cohorts for comparative analysis: one comprising patients newly initiated on beta-blocker therapy and the other including patients who were not, within days 1 to 7, after PCI for stable CAD with preserved LVEF. Beta-blocker therapy was defined as the treatment with metoprolol, carvedilol, atenolol, propranolol, and bisoprolol. Follow-up commenced on day 7 after PCI and ended at 5 years for both arms; regular checks ensured that patients remained free from beta-blocker exposure in the no beta-blocker arm throughout the study period. We determined treatment exposure for patients receiving beta-blocker therapy by checking patients' records at 90 days, 1 year, and 5 years after discharge.

We retrieved baseline characteristics from “day 1 prior to the index procedure up until any time before the procedure”; we performed propensity score matching, selecting a broad list of covariates, including demographics (age, sex, ethnicity/race), clinical comorbidities (body mass index, hypertension, diabetes, hyperlipidemia, smoking, prior stroke, prior MI, prior PCI, prior CABG, peripheral vascular disease, atrial fibrillation [AF]/flutter, prior HF, drug/alcohol abuse, chronic obstructive pulmonary disease, renal disease, neuropathy, and cancer), clinical presentation (heart rate and systolic blood pressure; dyspnea classified as the NYHA functional class [I-IV]), laboratory parameters (LVEF, low-density lipoprotein-cholesterol, hemoglobin, creatinine, and troponin), PCI setting (outpatient and inpatient), and medications at discharge (antiplatelet therapy, antihypertensives, antianginal, and lipid-lowering therapies) (Table 1).

**Table 1:** Baseline Characteristics of the Study Population After Propensity Matching

	Beta-Blocker (n = 11,681)	No Beta-Blocker (n = 11,681)	SMD
Age, y	74.0 (66.3-81.7)	73.9 (66.3-81.4)	0.008
Women	4,489 (38.4%)	4,529 (38.8%)	0.007
Ethnicity/race			
White adults	9,608 (82.3%)	9,573 (82.0%)	0.008
Black adults	1,399 (12.0%)	1,446 (12.4%)	0.012
Hispanic adults	549 (4.7%)	543 (4.6%)	0.002
Asian adults	176 (1.5%)	162 (1.4%)	0.01
Native American adults	35 (0.3%)	39 (0.3%)	0.006
Unknown	196 (1.7%)	202 (1.7%)	0.004
Comorbidities			
BMI ≥30 kg/m^2^	1,922 (8.8%)	1,804 (8.1%)	<0.001
Hypertension	10,832 (92.7%)	10,806 (92.5%)	0.009
Diabetes mellitus	7,316 (62.6%)	7,316 (62.6%)	<0.001
Hyperlipidemia	10,214 (87.4%)	10,195 (87.3%)	0.005
Smoking	4,619 (39.5%)	4,621 (39.6%)	<0.001
Prior stroke	2,620 (22.4%)	2,643 (22.6%)	0.005
Prior MI	5,758 (49.3%)	5,810 (49.7%)	0.009
Prior PCI	9,086 (77.8%)	9,086 (77.8%)	<0.001
Prior CABG	3,370 (28.9%)	3,428 (29.3%)	0.011
PVD	3,964 (33.9%)	3,971 (34.0%)	0.001
Atrial fibrillation/flutter	4,770 (40.8%)	4,820 (41.3%)	0.009
Prior heart failure	7,242 (62.0%)	7,407 (63.4%)	0.029
Drug abuse	1,760 (15.1%)	1,733 (14.8%)	0.006
Alcohol	1,050 (9.0%)	1,058 (9.1%)	0.002
COPD	4,002 (34.3%)	3,962 (33.9%)	0.007
Renal disease	5,348 (45.8%)	5,313 (45.5%)	0.006
Neuropathy	1,981 (17.0%)	1,965 (16.8%)	0.004
Cancer	1,372 (11.7%)	1,355 (11.6%)	0.005
Clinical presentation			
Median heart rate, beats/min	74 (62.0-85)	73 (61-85)	0.031
Median systolic BP, mm Hg	123 (106-139)	126 (111-142)	0.158
NYHA functional class I-II	357 (3.1%)	364 (3.1%)	0.003
NYHA functional class III-IV	330 (2.8%)	330 (2.8%)	<0.001
Laboratory values			
LVEF, %	58 (50-64)	57 (50-64)	0.096
LDL-C, mg/dL	82 (55-109)	82 (54-110)	0.001
Hgb, g/dL	11.3 (9.5-13.1)	12.2 (10.5-13.8)	0.355
Creatinine, mg/dL	1.5 (0.4-2.6)	1.4 (0.4-2.4)	0.063
Troponin, n/L	1.8 (0-9.5)	2.8 (0-11.7)	0.008
Setting			
Inpatient	3,832 (32.8)	3,820 (32.7)	<0.001
Outpatient	7,849 (67.2)	7,861 (67.2)	<0.001
Medications at discharge			
Aspirin	11,132 (95.3%)	11,132 (95.3%)	<0.001
P2Y12 inhibitor	8,393 (71.9%)	8,513 (72.9%)	0.023
ACEI/ARB	7,363 (63.0%)	7,458 (63.8%)	0.017
Statin	9,091 (77.8%)	9,117 (78.0%)	0.005
Fibrates	883 (7.6%)	886 (7.6%)	0.001
Ezetimibe	1,528 (13.1%)	1,540 (13.2%)	0.003
PCSK9 inhibitor	195 (1.7%)	202 (1.7%)	0.005
Diuretics agent	9,251 (79.2%)	9,375 (80.3%)	0.026
Calcium-channel blocker	8,451 (72.3%)	8,465 (72.5%)	0.003
Nitrates	9,305 (79.7%)	9,400 (80.5%)	0.02
Spironolactone	2,451 (11.2%)	2,385 (11.0%)	0.009
ARNI	2,622 (12.9%)	2,357 (10.8%)	0.03
SGLT2 inhibitor	1,245 (5.7%)	1,223 (5.6%)	0.004
Ranolazine	1,468 (6.7%)	1,328 (6.0%)	0.02

Values are median (Q1-Q3) or n (%).

ACEI = angiotensin-converting enzyme inhibitor; ARB = angiotensin receptor blocker; ARNI = angiotensin-neprilysin inhibitor; BMI = body mass index; CABG = coronary artery bypass graft surgery; COPD = chronic obstructive pulmonary disease; Hgb = hemoglobin; LVEF = left ventricular ejection fraction; LDL-C = low-density lipoprotein cholesterol; MI = myocardial infarction; PCI = percutaneous coronary intervention; PCSK9i = proprotein convertase subtilisin/kexin type 9 inhibitor; PVD = peripheral vascular disease; SGLT2 inhibitor = sodium-glucose co-transporter 2 inhibitor.

We employed an intention-to-treat analysis to preserve the benefits of the initial treatment assignment, whereby all patients who were started on beta-blockers within days 1 to 7 after PCI were included in the beta-blocker group for the entire duration of follow-up, regardless of adherence or discontinuation of therapy. Supplemental Table 2 reports the detailed protocol.

### Clinical endpoints

The primary endpoint was all-cause mortality. We focused on all-cause rather than cardiovascular mortality, given the difficult adjudication of causes of death. Secondary endpoints were hospitalization for MI, stroke, HF, and AF/flutter. Safety endpoints were hospitalization for bradycardia/second- or third-degree atrioventricular block, syncope, hypotension, pacemaker, and asthma/chronic obstructive pulmonary disease.

### Statistical analysis

We expressed continuous data as median values (Q1-Q3) and categorical variables as numbers (%). We performed the Rao-Scott chi-square test for categorical variables and the *t*-test or Wilcoxon rank-sum tests for continuous variables. Using logistic regression, we calculated the propensity score for the beta-blocker receipt vs no beta-blocker therapy. We incorporated a list of covariates (as above) in the propensity score model and applied a nearest neighbor greedy matching algorithm with a caliper width of 0.1 standard deviations of the logit of the propensity score.^13^ The balance achieved by the matching was assessed using standardized mean differences, with a value <0.1 indicating adequate balance (Table 1). Supplemental Figure 1 illustrates the propensity score density function before and after propensity matching.

We calculated incidence rates per 100 person-years of the outcomes for each exposure group, with 95% CIs based on the Poisson distribution. We used Kaplan-Meier estimates to plot the cumulative incidence of the outcomes for the study groups over the follow-up period. We fitted Cox proportional hazards regression models to estimate HRs and 95% CIs. All-cause mortality was treated as a competing risk when analyzing nonfatal outcomes. We used Fine and Gray's subdistribution hazard model^14^ to estimate subdistribution HRs for nonfatal outcomes, accounting for the competing risk of death. Participants were censored at the point any participant deviated from their assigned regimen.

We conducted several sensitivity analyses to further assess the association between beta-blockers and primary outcome. First, we utilized a bootstrap variance estimator with 1,000 bootstrap resamples as an alternative method to calculate the CIs of HR of the primary outcome (see Supplemental Methods).^15^ Second, we employed hospitalization for bone fracture and acute appendicitis as falsification endpoints, which are outcomes unrelated to the study's exposure or treatment. The absence of an association between these endpoints and the exposure lends credence to the unbiased relationship between the treatment and the primary endpoint.^16^ Third, we evaluated any potential effect of unmeasured confounding using the E-value methodology.^17^ This method estimates the minimum strength of association required between an unmeasured confounder and both receipt of beta-blocker therapy and risks of outcomes to overcome the statistically significant effect observed in a study where residual confounding is a potential problem. Fourth, we evaluated outcomes after excluding patients with AF and a prior history of HF. Fifth, we excluded patients who underwent inpatient PCI for stable CAD. Sixth, we changed the outcome assessment window starting 90 days after the PCI (to account for early post-PCI mortality potentially unrelated to beta-blocker therapy). Finally, we performed a per-protocol analysis, comparing outcomes between continuous beta-blocker users (on treatment exposure) and those who never used beta-blockers during the follow-up.

The statistical significance was set at *P* value <0.05 (2-sided). All statistical tests were conducted within the TriNetX Analytics Platform, and using R version 4.0.2 (R Foundation for Statistical Computing) and Python version 3.7 (Python Software Foundation).

## Results

### Characteristics of the patients

**Figure 1:**
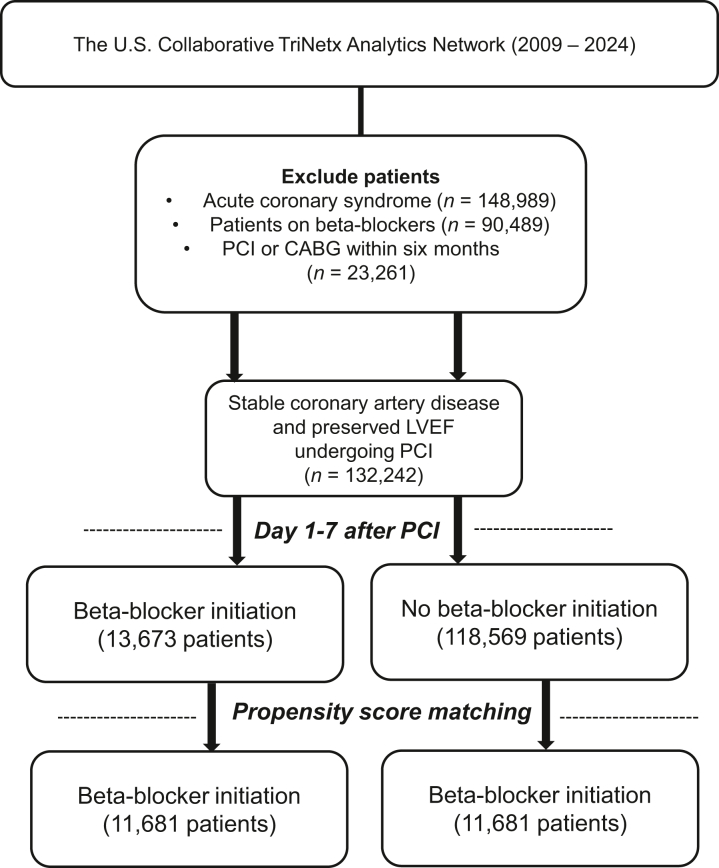
**Study Profile** CABG = coronary artery bypass graft; HF = heart failure; LVEF = left ventricular ejection fraction; PCI = percutaneous coronary intervention.

Between January 2009 and June 2024, we identified 132,242 adults who underwent PCI for stable CAD and preserved LVEF (Figure 1). Of these, 13,673 patients were initiated on beta-blocker therapy compared to 118,569 who did not receive beta-blockers. After propensity score matching, each group included 11,681 patients (Table 1). Supplemental Table 3 reports baseline characteristics before matching.

The median age of the patients was 74 years; 38.6% were women, and 82.1% were White adults. Regarding comorbidities, 92.6% of the patients had hypertension, 62.6% had diabetes mellitus, 87.4% had hyperlipidemia, and 49.5% had prior MI. About 77.8% had prior PCI, 29.1% had CABG, and 41.1% had a history of AF/flutter. Overall, 67.2% of patients underwent PCI in an outpatient setting, while 32.8% had the procedure during the inpatient stay. At discharge, 95.3% of the patients were prescribed aspirin, 72.4% a P2Y12 inhibitor, 63.4% an angiotensin-converting enzyme inhibitor or angiotensin receptor blocker, and 77.9% a statin.

Of 11,681 patients who had been assigned to the beta-blocker initiation group, 75% were treated with metoprolol, 15% with carvedilol, 8.5% with atenolol, and <2% with other agents. A total of 95% were reported to be on beta-blocker therapy at 90 days and 91% at 1 year after discharge.

### All-cause mortality

**Figure 2:**
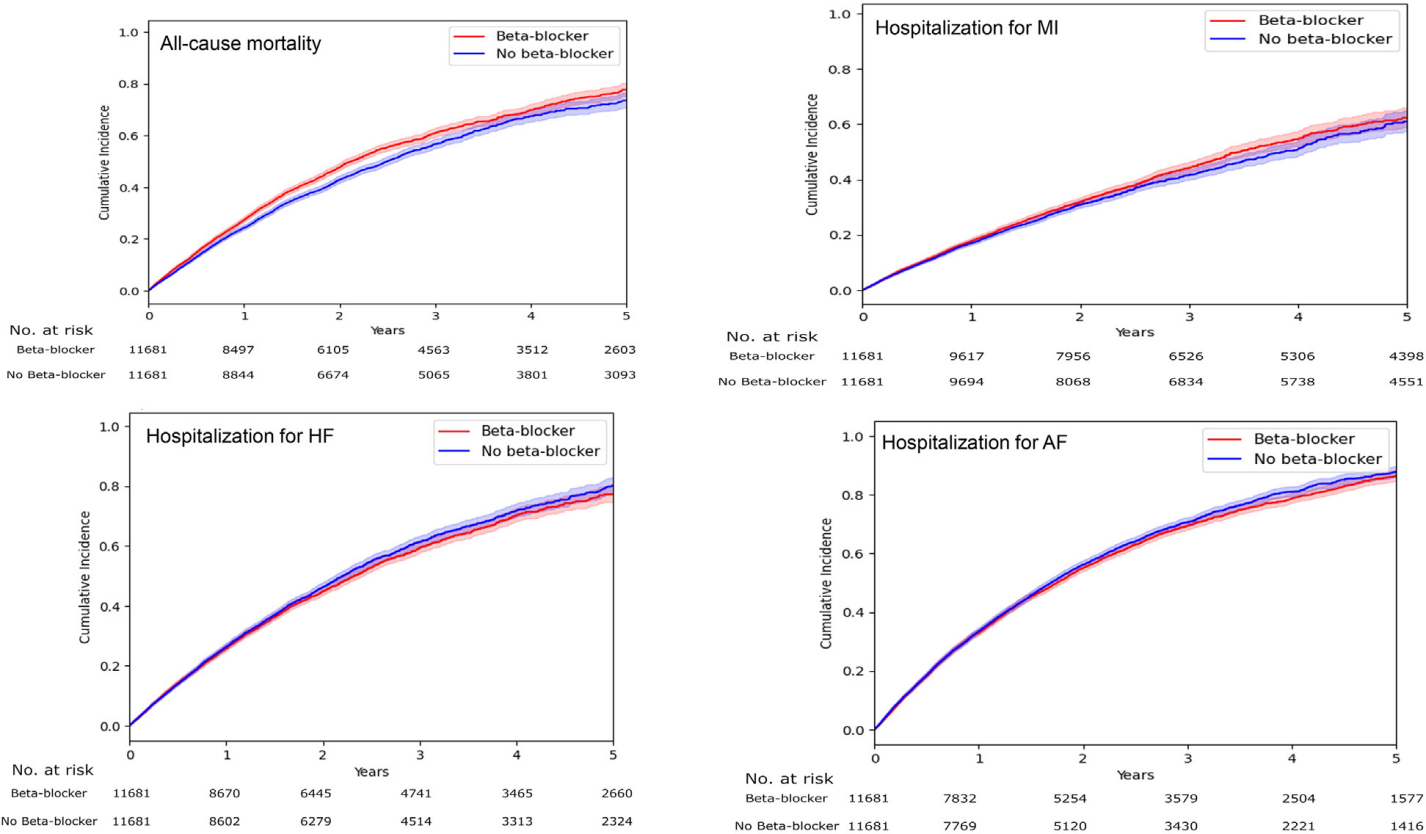
**Kaplan-Meier Plot Demonstrating the Effect of Beta-Blocker Therapy on All-Cause Mortality, Hospitalization for Myocardial Infarction, Heart Failure, and Atrial Fibrillation** AF = atrial fibrillation; HF = heart failure; MI = myocardial infarction.

A total of 3,664 of 11,681 patients died in the beta-blocker group, compared to 3,223 of 11,681 patients in the no beta-blocker group (Table 2). Beta-blocker therapy was associated with an increased risk of all-cause mortality (6.3 vs 5.5 per 100 person-years; HR: 1.11 [95% CI: 1.09-1.18]) (Figure 2).

**Table 2:** Five-Year Outcomes as per Intention to Treat Analysis

	Event		HR (95% CI)
	Incidence Rate (95% CI) per 100 Person-Years		
	Beta-Blocker (n = 11,681)	No Beta-Blocker (n = 11,681)	
All-cause mortality	3,664	3,223	1.11 (1.09-1.18)
	6.27 (6.25-6.29)	5.52 (5.50-5.54)	
Hospitalization for myocardial infarction	2,289	2,178	1.03 (0.97-1.09)
	3.92 (3.90-3.94)	3.73 (3.71-3.74)	
Hospitalization for stroke	1,132	1,144	0.98 (0.91-1.05)
	1.94 (1.93-1.96)	1.96 (1.95-1.97)	
Hospitalization for heart failure	3,510	3,551	0.99 (0.95-1.03)
	6.01 (5.92-6.03)	6.08 (6.06-6.10)	
Hospitalization for atrial fibrillation/flutter	4,730	4,799	0.97 (0.93-1.01)
	8.10 (8.08-8.12)	8.22 (8.19-8.24)	
Hospitalization for bradycardia/second- or third-degree atrioventricular block	3,976	4,006	0.98 (0.95-1.02)
	6.49 (6.47-6.51)	6.75 (6.72-6.77)	
Hospitalization for syncope	3,790	3,940	0.97 (0.94-1.02)
	6.49 (6.47-6.51)	6.75 (6.72-6.77)	
Hospitalization for hypotension	3,961	3,567	1.10 (1.06-1.14)
	6.78 (6.76-6.80)	6.11 (6.09-6.13)	
Hospitalization for pacemaker	1,563	1,690	0.95 (0.89-1.02)
	2.68 (6.66-2.69)	2.89 (2.88-2.91)	
Hospitalization for asthma/chronic obstructive pulmonary disease	1,516	1,455	1.03 (0.96-1.10)
	2.60 (2.58-2.61)	2.49 (2.48-2.50)	

Nonfatal cardiovascular outcomes were adjusted for competing risk of all-cause mortality.

### Secondary endpoints

There were no significant associations between beta-blocker treatment and hospitalization for MI (3.9 vs 3.7 per 100 person-years; HR: 1.03 [95% CI: 0.97-1.09]), HF (6.0 vs 6.1 per 100 person-years; HR: 0.99 [95% CI: 0.95-1.03]), AF/flutter (8.1 vs 8.2 per 100 person-years; HR: 0.97 [95% CI: 0.93-1.01]) (Figure 2), or stroke (1.9 vs 1.9 per 100 person-years; HR: 0.98 [95% CI: 0.91-1.05]) (Table 2).

### Safety endpoints

The rates of safety endpoints were also similar between both groups, except the hospitalization for hypotension was significantly higher with the beta-blockers (6.7 vs 6.1 per 100 person-years; HR: 1.10 [95% CI: 1.06-1.14]) (Table 2).

### Sensitivity analyses

The results were consistent for all-cause mortality using a bootstrap variance estimator (HR: 1.11 [95% CI: 1.05-1.17]). Beta-blockers showed no association with hospitalization for bone fracture (0.4 vs 0.4 per 100 person-years; HR: 1.02 [95% CI: 0.85-1.22]) or acute appendicitis (1.6 vs 1.4 per 100 person-years; HR: 1.17 [95% CI: 0.95-1.45]). The E-value for all-cause mortality was 1.46 (lower bound CI: 1.40), indicating that notable unmeasured confounding would overturn the observed association. Beta-blocker therapy was associated with a higher risk of all-cause mortality after excluding patients with AF and those with a history of HF (HR: 1.76 [95% CI: 1.47-2.12]), those who underwent inpatient PCI (HR: 1.31 [95% CI: 1.24-1.37]), and when follow-up commenced at 90 days after PCI (HR: 1.39 [95% CI: 1.24-1.56]). Finally, the outcomes were consistent in the per-protocol analyses, demonstrating a higher risk of all-cause mortality (6.4 vs 5.6 per 100 person-years; HR: 1.14 [95% CI: 1.09-1.18]) and no impact on cardiovascular outcomes with beta-blocker vs no beta-blocker therapy (Supplemental Table 4). Central Illustration shows study flow chart and main findings.

**Central illustration:**
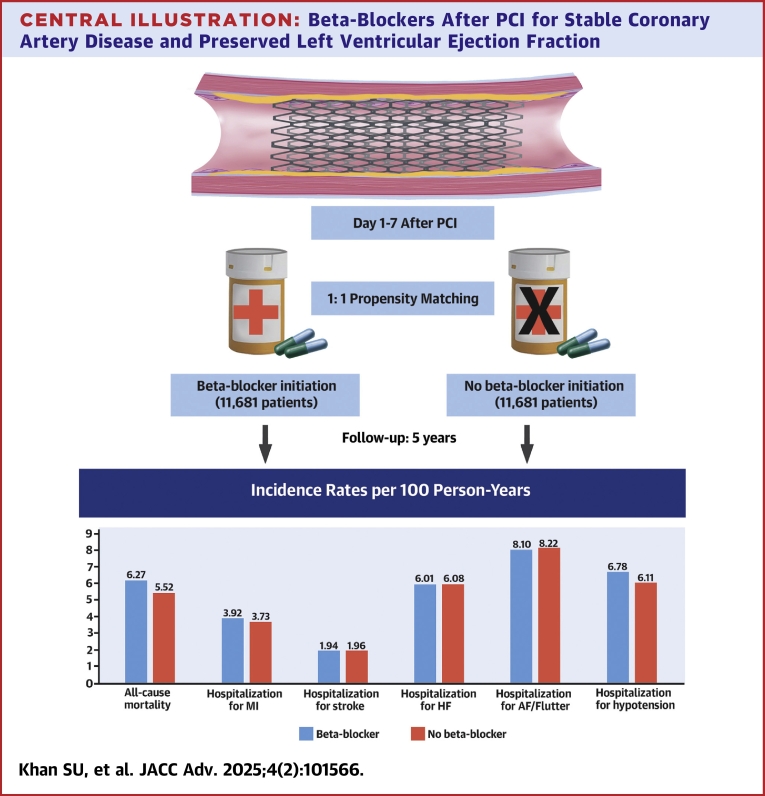
**Beta-Blockers After PCI for Stable Coronary Artery Disease and Preserved Left Ventricular Ejection Fraction** PCI = percutaneous coronary intervention; other abbreviations as in Figure 2.

## Discussion

The results of this population-based cohort study, designed to emulate a randomized controlled trial, suggest that the strategy of early initiating beta-blocker therapy after PCI for stable CAD and preserved LVEF was associated with higher all-cause mortality at 5 years compared to patients not initiated on beta-blockers. Beta-blocker initiation was not associated with lowering the risk of MI, stroke, HF, and AF/flutter hospitalizations. Furthermore, beta-blockers were associated with a safety concern related to hospitalization for hypotension. These results remained consistent across several sensitivity analyses, including after excluding patients with AF and prior HF and patients who underwent inpatient PCI.

A Canadian study demonstrated a reduction in a composite of all-cause mortality, HF, or MI hospitalization with beta-blocker therapy in patients with angiographically documented stable CAD without HF.^4^ However, that study defined the beta-blocker use based on the prescription claims either 90 days before or after the index coronary angiography, with the outcome assessment window beginning 90 days after the index coronary angiography. In contrast, we focused on patients who underwent PCI for stable CAD with preserved LVEF and clearly defined the start of follow-up for both study groups from the treatment initiation, extending up to 5 years. This precise demarcation enhances reliability by minimizing immortal time bias^7^ and time-window bias.^18^

Our results are consistent with studies demonstrating potential harm with beta-blocker therapy in stable CAD. The REACH (Reduction of Atherothrombosis for Continued Health) showed higher rates of composite secondary (cardiovascular death, nonfatal MI, nonfatal stroke, hospitalization for atherothrombotic events, or revascularization) and tertiary (all-cause and cardiovascular mortality, nonfatal MI and stroke, and hospitalization) endpoints.^19^ In the NCDR (National Cardiovascular Data Registry) study, among 755,215 patients who underwent PCI for stable CAD (71.4% were discharged on beta-blockers), there were no reductions in the adjusted mortality, MI, or coronary revascularization rates; instead, there were a higher risk of HF hospitalization at 30 days and 3 years.^20^

These findings are particularly notable in light of the ABYSS (Assessment of Beta-Blocker Interruption 1 Year after an Uncomplicated Myocardial Infarction on Safety and Symptomatic Cardiac Events Requiring Hospitalization) trial, where stopping beta-blockers did not meet the noninferiority margin for the primary composite outcome of death, nonfatal MI, nonfatal stroke, or rehospitalization for cardiovascular reasons. However, the ABYSS trial noted that interrupting beta-blocker therapy did not improve patient-reported quality of life and led to increased hospitalizations for angina. This was particularly relevant for patients with a midrange LVEF (40% to 49%), where the ABYSS trial findings suggest a nuanced benefit that might differ from patients with higher LVEF.

Another recent study, the REDUCE-AMI (Randomized Evaluation of Decreased Usage of Beta-Blockers after Acute Myocardial Infarction) trial, showed no reduction in all-cause mortality or MI with beta-blocker therapy over a median follow-up of 3.5 years.^21^ These recent studies and our report collectively suggest that in the era of modern revascularization and medical therapy, the role of beta-blockers needs careful re-evaluation, especially in patients with preserved LVEF after PCI.

The observed increase in all-cause mortality could be attributed to several factors inherent to patients with stable CAD and preserved LVEF, where the pathophysiological benefits of reduced myocardial oxygen demand and HR control may not outweigh the risks of adverse inotropic effects and potential for hypotension. Moreover, the absence of left ventricular dysfunction might reduce the necessity and effectiveness of beta-adrenergic blockade in this subgroup, as the primary mechanisms of sudden cardiac death and myocardial ischemia differ from those with compromised cardiac function.^22^ Furthermore, in patients undergoing revascularization, beta-blockers are unlikely to provide further benefits after revascularization with normal LVEF, mainly when the patients are compliant with guideline-directed therapies.^23^ In a meta-analysis of 60 trials (102,003 patients), beta-blockers significantly reduced mortality and angina beyond 1 year in trials conducted in the pre-reperfusion era.^24^ However, during the reperfusion era, trials demonstrated beta-blocker benefits were limited to reducing MI and angina at 30 days, with notable increases in HF, cardiogenic shock, and drug discontinuation within the first year.^24^

The strength of the current study lies in adopting an incident user design and target trial emulation approach that may minimize several biases. Second, the TriNetX database, with many sites, geographical range, and diversity, allowed us to adjust to a wide range of potential confounders. While our study uniquely tracked continuous beta-blocker prescription status after discharge, a factor not considered in previous studies, patients' actual adherence to treatment regimens remains uncertain. However, the observed patterns mirror the adherence patterns in clinical trials and real-world settings.^21^^,^^25^^,^^26^ Finally, our conclusions are supported by the consistency of the results across multiple sensitivity analyses, coupled with a thorough evaluation of efficacy and safety profiles of the beta-blocker initiation strategy.

### Study Limitations

This observational study lacks randomization, making it challenging to establish causality. Despite employing propensity score matching, the potential for unmeasured confounding exists, particularly concerning variables that did not achieve balance through matching, as well as socioeconomic factors, lifestyle behaviors, and missing data on anatomical and procedural details from PCI. In addition, we could not assess the extent of CAD that may have been observed at the time of coronary angiography and PCI that might have prompted the initiation of beta-blocker therapy. On the same note, one-third of patients underwent PCI during the inpatient stay; one potential explanation is that some patients may have troponin elevation at baseline, prompting inpatient PCI. In the EVENT (Evaluation of Drug Eluting Stents and Ischemic Events) registry, 6% of patients who underwent PCI for stable CAD had elevated troponin at baseline.^27^ Furthermore, a significant proportion of patients with HF history suggests that we may have included patients with HF with preserved ejection fraction. However, our sensitivity analysis, excluding patients who underwent inpatient PCI and history of HF, showed consistent results. The reliance on Current Procedure Terminology and ICD coding systems also introduces the possibility of coding errors and misclassifications, although this is less likely to impact all-cause mortality.^21^ Finally, we focused on endpoints associated with hospitalization, which may not capture the entire spectrum of outcomes.

## Conclusions

The results of this population-based study, designed to emulate a randomized controlled trial, suggest that the strategy of early initiation of beta-blockade after PCI for stable CAD in patients with preserved left ventricular systolic function may not confer the anticipated cardiovascular benefits and could potentially increase the risk of all-cause mortality. These findings support the current professional guidelines in this patient population (Class of Recommendation III: No benefit), emphasizing the need for personalized therapeutic strategies based on individual risk profiles rather than a universal approach to prescribing beta-blockers.

## Funding support and author disclosures

The authors have reported that they have no relationships relevant to the contents of this paper to disclose.
