# Supplementary material for: Beta-Blockers After PCI for Stable Coronary Artery Disease and Preserved Left Ventricular Ejection Fraction
Source: JACC Adv. 2025 Jan 17;4(2):101566. doi: 10.1016/j.jacadv.2024.101566 (PMC11787421; doi:10.1016/j.jacadv.2024.101566)
Supplement: Supplemental Material [file mmc1.docx]

**SUPPLEMENTAL APPENDIX**

**Supplemental Methods**

We employed Kaplan-Meier survival curves for all-cause mortality to reconstruct time-to-event data using WebPlotDigitizer (Version 4.0). This tool allowed us to extract precise survival probabilities and at-risk numbers at specified time intervals, enabling accurate replication of the survival function. We generated a dataset from these data points with assigned event times—either to an event or to censoring—based on the survival probabilities and intervals from the Kaplan-Meier curves.

We applied a bootstrap resampling technique to enhance the robustness of our data analysis. For each of 1,000 bootstrap iterations, we randomly sampled with replacement from the dataset to create new samples of the same size. These samples were analyzed using Cox proportional hazards models to estimate median survival times and hazard ratios, with 95% confidence intervals derived from the 2.5th and 97.5th percentiles of the bootstrap distribution.

To estimate person-years, we digitized the Kaplan-Meier curves using WebPlotDigitizer, calculating midpoint survival probabilities for defined time intervals and then estimating the average person-time contribution per interval. The total person-years for each group were then computed by summing these interval contributions and multiplying by the total number of patients initially at risk.

All analyses were conducted using R statistical software (version 4.0.2, R Foundation for Statistical Computing, Vienna, Austria), utilizing the `survival` package for survival analyses and the `boot` package for bootstrap resampling procedures.

**Supplemental Table 1.** ICD-10 codes.

| **Label** | **ICD-10** |
| --- | --- |
| Percutaneous coronary intervention. | 02713ZZ, 02723ZZ, 02723ZZ, 027034Z, 02713DZ, 02723DZ, 02733DZ, 02703DZ, 02713ZZ, 02723ZZ, 02733ZZ, Z98.61, 1021163 |
| Coronary artery disease | I25 |
| All-cause mortality | Deceased |
| Myocardial infarction | I21.x, I22.x |
| Heart failure hospitalization | I50.x |
| Stroke | I60-64 |
| Atrial fibrillation hospitalization | I48, I48.91, I48.9 |
| Bradycardia | R00.1, I49.5 |
| AV block II-III | I44.1-3 |
| Hypotension | I95 |
| Syncope | R55.9, T67.1 |
| Need for pacemaker | FPE00-26, FPF00-20, TFP00 |
| Asthma hospitalization | J45-46 |
| COPD hospitalization | J44 |
| Bone fracture | S02.0, S42.0, S42.2, S52.5, S52.6, S72.0, S82.1, S82.2, S82.8, S22.3 |
| Acute appendicitis | K35.80 |

**Supplemental Table 2.** Comparison between target trial and emulated trial using real-world data.

| **Components** | **Target trial** | **Emulated trial using RWD** |
| --- | --- | --- |
| Primary objective | - To assess whether strategy of early initiating oral beta-blockade in patients with stable coronary artery disease and preserved left ventricular (LV) systolic function who underwent percutaneous coronary intervention (PCI) reduces all-cause mortality. - Focus on all-cause rather than cardiovascular mortality given the difficult adjudication of causes of death and given relatively low mortality expected. | - To assess whether strategy of early initiating oral beta-blockade in patients with stable coronary artery disease and preserved left ventricular (LV) systolic function who underwent percutaneous coronary intervention (PCI) reduces all-cause mortality. - Focus on all-cause rather than cardiovascular mortality given the difficult adjudication of causes of death and given relatively low mortality expected. |
| Secondary objective | To assess whether strategy of early initiating of oral beta-blockade in patients with stable coronary artery disease and preserved LV systolic function who underwent percutaneous coronary intervention (PCI) reduces the risk of hospitalization for:   - Myocardial infarction - Stroke - Heart failure - Atrial fibrillation/flutter | To assess whether strategy of early initiating of oral beta-blockade in patients with stable coronary artery disease and preserved LV systolic function who underwent percutaneous coronary intervention (PCI) reduces the risk of hospitalization for:   - Myocardial infarction - Stroke - Heart failure - Atrial fibrillation/flutter |
| Tertiary objective | To assess whether strategy of early initiating oral beta-blockade in patients with stable coronary artery disease and preserved LV systolic function who underwent percutaneous coronary intervention (PCI) influences the risk of hospitalization for:   - Bradycardia/AV II-III blocks - Syncope - Hypotension - Need for pacemaker. - Asthma/COPD | To assess whether strategy of early initiating oral beta-blockade in patients with stable coronary artery disease and preserved LV systolic function who underwent percutaneous coronary intervention (PCI) influences the risk of hospitalization for:   - Bradycardia/AV II-III blocks - Syncope - Hypotension - Need for pacemaker. - Asthma/COPD |
| Inclusion criteria | - Men or women age 18+ years at the time of signing the informed consent. - Day 1 to 7 after PCI for stable coronary artery disease - Coronary angiography performed during hospitalization. - Obstructive coronary artery disease documented by coronary angiography, i.e. stenosis 50%+, FFR ≤ 0.80 or iFR ≤0.89 in any segment at any time before randomization. - Echocardiography performed after the PCI showing normal ejection fraction defined as EF ≥50%. | - Same with assumption that individuals who received PCI would have agreed to participate in the study and had coronary angiography demonstrating obstructive coronary artery disease documented by coronary angiography, i.e. stenosis 50%+, FFR ≤ 0.80 or iFR ≤0.89 in any segment. - Echocardiography performed after the PCI showing normal ejection fraction defined as EF ≥50%. |
| Exclusion criteria | -Any condition that influences the patient’s ability to comply with study protocol.  -Patients with acute coronary syndrome  - Revascularization by CABG within six months or patients treated with medical therapy alone during hospitalization  -Revascularization by PCI within six months.  - Contraindication for beta-blockade  - Indication for beta-blockade other than secondary prevention, according to the treating physician. | - Patients with acute coronary syndrome  - Revascularization by CABG within six months or patients treated with medical therapy alone during hospitalization  --Revascularization by PCI within six months.  - Exclusion of patients already on  baseline beta-blockers with an assumption that beta-blockade was exclusively initiated for secondary prevention after PCI. |
| Treatment assignment | Patients after PCI for stable coronary artery disease were randomly assigned (between day 1 and 7) to start on beta-blocker therapy vs. no beta-blocker therapy. | Patients were initiated on beta-blocker therapy vs. no beta-blocker therapy (between day 1 and 7) of PCI for stable coronary artery disease. Randomization is emulated by matching on a range of covariates measured at the time of initiation of therapy. |
| Follow-up | Follow-up starts at treatment assignment and ends at 5 years. | Follow-up starts on the date of initiation of therapy and ends at 5 years. |
| Outcomes | Efficacy and safety outcomes | Effectiveness and safety outcomes |
| Causal contrast | Intention-to treat effects | Intention-to treat effects |
| Statistical analysis | 1. Intention to treat analysis 2. Per Protocol analysis | 1. Intention to treat analysis with propensity score matching for various covariates. 2. Per Protocol analysis |

**Supplemental Table 3**. Baseline characteristics of the study population before propensity matching.

| **Characteristics** | **Beta-blocker**  **(*n* = 13,673)** | **No beta-blocker**  **(*n* = 118,569)** |
| --- | --- | --- |
| Age, years | 73.9 (66.27-81.53) | 73.6 (65.91-81.30) |
| Women | 5,327 (39.0%) | 39,928 (33.7%) |
| **Ethnicity/race** |  |  |
| White adults | 11,158 (81.6%) | 100,347 (84.6%) |
| Black adults | 1,739 (12.7%) | 8,496 (7.2%) |
| Hispanic adults | 698 (5.1%) | 4,567 (3.9%) |
| Asian adults | 199 (1.5%) | 2,214 (1.9%) |
| *Native American adults | 44 (0.3%) | 299 (0.3%) |
| Unknown | 212 (1.6%) | 2,941 (2.5%) |
| **Comorbidities** |  |  |
| BMI ≥ 30 kg/m2 | 1910 (8.8%) | 3755 (4.0%) |
| Hypertension | 12,772 (93.4%) | 93,682 (79.0%) |
| Diabetes mellitus | 8,906 (65.1%) | 49,249 (41.5%) |
| Hyperlipidemia | 12,115 (88.6%) | 85,781 (72.4%) |
| Smoking | 5,711 (41.8%) | 29,753 (25.1%) |
| Prior stroke | 3,320 (24.3%) | 12,747 (10.8%) |
| Prior MI | 7,081 (51.8%) | 35,206 (29.7%) |
| Prior PCI | 11,076 (81.0%) | 54,686 (46.1%) |
| Prior CABG | 4,161 (30.4%) | 19,919 (16.8%) |
| PVD | 5,029 (36.8%) | 21,179 (17.9%) |
| Atrial fib/flutter | 5,880 (43.0%) | 29,143 (24.6%) |
| Prior heart failure | 8,972 (65.6%) | 38,511 (32.5%) |
| Drug abuse | 2,288 (16.7%) | 8,439 (7.1%) |
| Alcohol | 1,369 (10.0%) | 5,490 (4.6%) |
| COPD | 5,045 (36.9%) | 22,143 (18.7%) |
| Renal disease | 6,672 (48.8%) | 28,474 (24.0%) |
| Neuropathy | 2,551 (18.7%) | 10,006 (8.4%) |
| Cancer | 1,748 (12.8%) | 6,262 (5.3%) |
| **Clinical presentation** |  |  |
| Median heart rate, beats/min | 74 (62-86) | 72 (61-84) |
| Median SBP, mmHg | 122 (106-139) | 127 (112-143) |
| NYHA class I-II | 575 (4.2%) | 1,161 (1.0%) |
| NYHA class III-IV | 549 (4.0%) | 882 (0.7%) |
| **Laboratory values** |  |  |
| LVEF, (Q1-Q3), % | 58 (50-64) | 56 (50-64) |
| LDL-C, mg/dL | 81 (55-108) | 87 (60-114) |
| Hgb, g/dL | 11.2 (9.5-12.8) | 12.9 (11.3-14.4) |
| Creatinine, mg/dL | 1.5 (0.4-2.2) | 1.2 (0.32-2.1) |
| Troponin, n/L | 1.9 (0-9.3) | 3.0 (0 - 15.7) |
| **Medications at discharge** |  |  |
| Aspirin | 13,117 (95.9%) | 87,134 (73.5%) |
| P2Y12 inhibitor | 10,109 (73.9%) | 58,202 (49.1%) |
| ACEI/ARB | 8,863 (64.8%) | 51,986 (43.9%) |
| Statin | 10,889 (79.6%) | 65,790 (55.5%) |
| Fibrates | 1,053 (7.7%) | 5,950 (5.0%) |
| Ezetimibe | 1,865 (13.6%) | 11,640 (9.8%) |
| PCSK9 inhibitor | 248 (1.8%) | 1,195 (1.0%) |
| Diuretics agent | 11,184 (81.8%) | 55,302 (46.7%) |
| Calcium-channel blocker | 10,238 (74.9%) | 52,664 (44.4%) |
| Nitrates | 11,518 (84.2%) | 70,220 (59.2%) |
| Spironolactone | 2457(11.28%) | 7645 (8%) |
| ARNI | 2625 (12%) | 8460 (9%) |
| SGLT2 inhibitor | 1248 (5.73%) | 3712 (3.9%) |
| Ranolazine | 1474 (6.76%) | 3227 (3.4%) |

All variables are statistically significant (P<0.05) except *.

ARNI: Angiotensin-Neprilysin Inhibitor; ACEI: angiotensin-converting enzyme inhibitor; ARB: angiotensin receptor blocker; BMI: body mass index; CABG: coronary artery bypass graft surgery; Hgb: hemoglobin; LVEF: left ventricular ejection fraction; LDL-C: low density lipoprotein-cholesterol; NYHA: New York Heart Association; MI: myocardial infarction; PCSK9i: Proprotein convertase subtilisin/kexin type 9 inhibitor; PCI: percutaneous coronary intervention; PVD: peripheral vascular disease; SBP: systolic blood pressure; SGLT2 inhibitor: Sodium-Glucose Co-Transporter 2 inhibitor.

**Supplemental Table 4**. Five-year outcomes as per protocol analysis.

|  | **Event** | | **Hazard ratio**  **(95% CI)** |
| --- | --- | --- | --- |
|  | **Incidence rate (95% CI) per 100 person-years** | |  |
| **Outcome** | **Beta-blocker** | **No beta-blocker** |  |
| All-cause mortality | 3,664/11,372 | 3,223/11,372 | 1.14 (1.09, 1.18) |
|  | 6.44 (6.42, 6.46) | 5.67 (5.65, 5.69) |  |
| Hospitalization for myocardial infarction | 2289/11,372 | 2178/11,372 | 1.03 (0.97, 1.09) |
|  | 4.03 (4.01, 4.04) | 3.83 (3.81, 3.85) |  |
| Hospitalization for stroke | 1,132/8,124 | 1,144/8,355 | 1.01 (0.93, 1.09) |
|  | 2.79 (2.77, 2.80) | 2.74 (2.72, 2.75) |  |
| Hospitalization for heart failure | 3,510/11,372 | 3,551/11,372 | 0.97 (0.92, 1.02) |
|  | 6.17 (6.15, 6.19) | 6.25 (6.22, 6.27) |  |
| Hospitalization for atrial fibrillation/flutter | 4,730/11,626 | 4,799/11,626 | 0.98 (0.95, 1.01) |
|  | 8.14 (8.11, 8.16) | 8.26 (8.23, 8.28) |  |
| Hospitalization for bradycardia/ second- or third-degree atrioventricular block | 3,976/11,681 | 4,006/11,681 | 0.97 (0.94, 1.02) |
|  | 6.81 (6.79, 6.83) | 6.86 (6.84, 6.88) |  |
| Hospitalization for syncope | 3,790/11,681 | 3,940/11,681 | 0.95 (0.92, 1.00) |
|  | 6.49 (6.47, 6.51) | 6.75 (6.72, 6.77) |  |
| Hospitalization for hypotension | 3,961/11,681 | 3,567/11,681 | 1.09 (1.05, 1.13) |
|  | 6.78 (6.76, 6.80) | 6.11 (6.09, 6.13) |  |
| Hospitalization for pacemaker | 1,563/11,681 | 1,690/11,681 | 0.95 (0.89, 1.02) |
|  | 2.68 (6.66, 2.69) | 2.89 (2.88, 2.91) |  |
| Hospitalization for Asthma/chronic obstructive pulmonary disease | 1,563/11,681 | 1,690/11,681 | 1.01 (0.95, 1.08) |
|  | 2.68 (2.66, 2.69) | 2.89 (2.88, 2.91) |  |

Non-fatal cardiovascular outcomes were adjusted for competing risk of all-cause mortality.

**Supplemental Figure 1.** Propensity score density function before and after matching.
